# The Nuclear Translocation of Heme Oxygenase-1 in Human Diseases

**DOI:** 10.3389/fcell.2022.890186

**Published:** 2022-06-29

**Authors:** Qing Yang, Wenqian Wang

**Affiliations:** ^1^ Department of Breast Surgery, The Second Affiliated Hospital and Yuying Children’s Hospital of Wenzhou Medical University, Wenzhou, China; ^2^ Department of Plastic Surgery, The Second Affiliated Hospital and Yuying Children’s Hospital of Wenzhou Medical University, Wenzhou, China

**Keywords:** heme oxygenase-1, nuclear translocation, noncanonical function, tumorigenesis, inflammation, anticancer therapy

## Abstract

Heme oxygenase-1 (HO-1) is a rate-limiting enzyme in the degradation of heme to generate carbon monoxide (CO), free iron and biliverdin, which could then be converted to bilirubin by biliverdin reductase. HO-1 exhibits cytoprotective effects of anti-apoptosis, anti-oxidation, and anti-inflammation *via* these byproducts generated during the above process. In the last few years, despite the canonical function of HO-1 and possible biological significance of its byproducts, a noncanonical function, through which HO-1 exhibits functions in diseases independent of its enzyme activity, also has been reported. In this review, the noncanonical functions of HO-1 and its translocation in other subcellular compartments are summarized. More importantly, we emphasize the critical role of HO-1 nuclear translocation in human diseases. Intriguingly, this translocation was linked to tumorigenesis and tumor progression in lung, prostate, head, and neck squamous cell carcinomas and chronic myeloid leukemia. Given the importance of HO-1 nuclear translocation in human diseases, nuclear HO-1 as a novel target might be attractive for the prevention and treatment of human diseases.

## Introduction

Heme oxygenase-1 (HO-1) is the inducible subtype of HO, which is the crucial rate-limiting enzyme that catalyzes the degradation of heme (MD, 1997; Bauer et al., 1998). Under basal conditions, HO-1 is constitutively expressed in human spleen and liver (Sugishima et al., 2000). However, abnormal or stress states can significantly upregulate HO-1 to degrade heme and produce bile pigments as well as carbon monoxide (CO), which are essential antioxidants and signaling molecules, thus maintaining cellular homeostasis (Md and Pe, 2005; Gozzelino et al., 2010; Loboda et al., 2015). It is designated as the canonical function of HO-1, which is related to its enzymatic activity for heme degradation, and has been studied in many physiological and pathological situations.

In recent years, a growing number of papers are adding insights into the noncanonical function of HO-1. It contains the signaling function of an inactive form of HO-1 proteins in the cytoplasm and the function arising from HO-1 localization in both nuclear and cytoplasm compartments, such as mitochondria and caveolae, where localization in nuclei is particularly important (Dennery 2014). Nuclear HO-1 protein expression is detectable in cultured cells with increased exposure to stressful conditions, and this localization is probably associated with upregulation of genes that improve cell protection from oxidative stress (Lin et al., 2007). Recently, growing studies have revealed aberrant HO-1 accumulation in the nucleus of tumor cells, and antitumor therapy could further upregulates nuclear HO-1 expression (Sacca et al., 2007; Li et al., 2008; Degese et al., 2012; Gandini et al., 2012; Tibullo et al., 2013; Dennery 2014). These findings could open an exciting new area of cancer research, namely that HO-1 nuclear translocation is relevant to tumor progression and resistance to treatment. However, the exact mechanisms of modulation of human diseases by nuclear HO-1 remains to be elucidated. In this review, the significance of nuclear HO-1 expression in different types of diseases and critical structure for the translocation of HO-1 to nuclei are discussed in detail. Furthermore, we elaborate on several signaling pathways in which nuclear HO-1 may be involved.

### Structure and Function of HO-1

**FIGURE 1:**
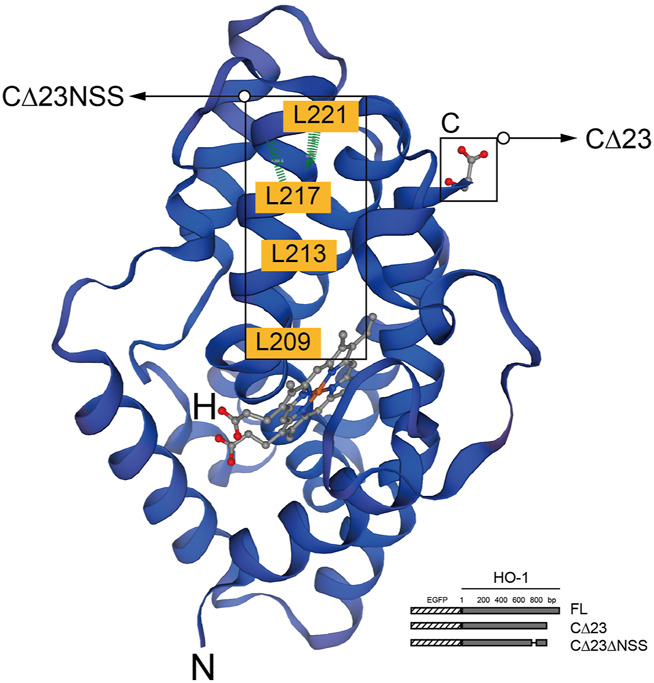
Structure of HO-1 protein generated by the SWISS-MODEL tool. The highlighted region is C-terminal, which is critical for protein to be anchored in the smooth endoplasmic reticulum(sER), and the region abundant in leucine homologous to NES motif. The binding domain of heme is marked in red. Schematic representation of HO-1 cDNA obliteration and truncation are shown in the lower right corner. Hatched section, EGFP; solid section, HO-1 cDNA mutants. FL, full-length HO-1 cDNA; CΔ23, HO-1 cDNA without the C-terminal 23 amino acids; CΔ23ΔNSS, HO-1 cDNA without the C-terminal 23 amino acids and the NSS sequence.

In humans, HO-1 is generated from the full-length mRNA transcript of the *HMOX1* gene, which is located on chromosome 22q13.3 and has five exons and four introns and encodes a 32 kDa molecular weight protein with 288 amino acids (Yoshinaga et al., 1982b). The HO-1 is normally transported to and attached at the smooth endoplasmic reticulum (sER) membrane by a C-terminal transmembrane segment (Shibahara et al., 1985). Under stress or pathological conditions, HO-1 was found to be localized in other subcellular compartments, including the plasma membrane, the mitochondria, and the nucleus (Dunn et al., 2014). HO-1 nuclear translocation is known to be sensitive to cellular responses, particularly oxidative stress (Mascaró et al., 2021). There are two different nuclear forms of HO-1, 32 kDa complete form and 28 kDa C-terminal truncated HO-1 form, which is the main nucleus form (Yoshida et al., 1991; Lin et al., 2007) (Figure 1).

### HO-1 Nuclear Localization and Oxidative Stress

Oxidative stress is a pro-oxidative condition that occurs when there is an imbalance between oxidants and antioxidants favoring the oxidants, and has been linked to both normal physiological and pathological processes. When the production of reactive oxygen species (ROS) exceeds the ability of intrinsic antioxidants and antioxidative defenses to neutralize them, oxidative stress occurs. Intracellular ROS are produced primarily by the mitochondrial electron transport chain, NADPH oxidases, and xanthine oxidase, and also triggered by external factors such as electrophiles and UV radiation (Chiang et al., 2021). The upregulation of cellular HO-1 expression is an indicator of oxidative stress, because of its downstream metabolites, such as anti-oxidant biliverdin and pro-oxidant ferrous iron (Tenhunen et al., 1972; Ryter et al., 2016). It has been shown that HO-1 protein could bind to and transport the Nrf2 complex, which migrates to the nucleus, which might promote the stabilization of the Nrf2 by the Akt/GSK3 and PI3K genes (Rada et al., 2012; Chowdhry et al., 2013; Biswas et al., 2014). In addition, nuclear HO-1 could also participate in the protection against Nrf2-mediated oxidation by inducing mRNA expression of G6PDH and NQO1 (Biswas et al., 2014).These studies demonstrated that the nuclear localization of HO-1 could also contributes to the protection against oxidative stress by upregulating the expression of antioxidant genes.

### Nuclear Protein Import Machinery

HO-1, a type II membrane protein, is an integral membrane protein immobilized on the sER through a short carboxyl-terminal transmembrane segment (TMS), which contains 23 amino acids and resides in the lumen of sER (Yoshinaga et al., 1982a). HO-1 nuclear translocation involves the hydrolyzation of the transmembrane (TM) domain and the release of large HO-1 fragments which contain the N terminus in cytosol (Lin et al., 2007). Previous studies revealed that mutations within TMS degraded HO-1, W270N, hindering the oligomerization of HO-1 and making it more suitable for protein cleavage (Hwang et al., 2009), suggesting that HO-1 is sensitive to proteolysis and may undergo intramembrane proteolysis, leading to nuclear localization (Yoshida et al., 1991). Signal Peptide Peptidase (SPP) is a heterogeneous enlarged membrane protein presents in the endoplasmic reticulum and one of five SPPs marked in mammals, which catalyzes the intramembrane cleft of membrane type II proteins and promotes the catalysis of proteolytic HO-1 and its nuclear translocation *in vitro* (Fluhrer et al., 2009). The TMS in HO-1 constitutes the α-helix shape which is similar to a hydrophobic central region peptide signal (Erez et al., 2009; Hwang et al., 2009). SPP was found to hydrolyze HO-1 after residues of S275 and F276, which contain high α-helical potential within TMS (Pace and Scholtz 1998). This finding shares the similarity to previous reports demonstrating that SPP induces cleavage in membranes at different sites (Okamoto et al., 2008; Fluhrer et al., 2009). In A549 lung cancer cells and DU145 prostate cancer cells that overexpression of HO-1 and SPP, knockdown of the SPP genes using siRNA or pharmacologic inhibition of SPP significantly reduced nuclear localization of HO-1 (Hsu et al., 2015). And these results are consistent with those obtained *in vitro* experiments (Yoshida et al., 1991). This indicates that proteolytic cleavage is essential for carboxyl-terminal truncation and HO-1 nuclear localization (Lin et al., 2007). After being unleashed from the sER *via* C-terminal truncation, HO-1 could translocate to the nucleus and play a non-canonical role. There are currently few studies on how nuclear HO-1 enters and exits the nuclear compartment.

Protein nuclear translocation could exist *via* passive diffusion if molecular weights of proteins below 50 kDa or *via* active transport if there is nuclear localization sequences (NLSs) of proteins (Schlenstedt 1996; Macara 2001). The nuclear pore complex is a basket-like complex structure embedded in the inner and outer nuclear membranes, which is the channel for substances to enter and exit the nucleus (Ryan and Wente 2000). The machinery of macromolecules translocated into nuclei relies on energy and carrier by active transport (Conti and Izaurralde 2001). Karyopherin binds to NLS to form a pore-targeting complex which docks at the distal end of the fibrils protruding from the cytoplasmic ring of the nuclear pore complex, after which it traverses the pore in an energy-dependent way that has not yet been fully acknowledged (Escriou et al., 2003). Although bioinformatic study has identified a monopartite NLS at position 111 and a projected bipartite NLS at position 196 for HO-1 protein (Vanella et al., 2016), whether an importin-related mechanism is involved in nuclear HO-1 import needs further to be investigated. There are also other possibilities that the truncated form of HO-1 forms a complex with other cytoplasmic proteins that possess an NLS (Weng et al., 2004), or interacting with other proteins to facilitate its nuclear translocation (Li Volti et al., 2004).

### Nuclear Localization Regions

According to previous report, similar to HIV-Rev NLS, the highly conserved leucine-rich domain as the putative nuclear shuttling sequence (NSS) motif was found near the C-terminal of HO-1 (Henderson and Percipalle 1997). Transfection of mutants missing the 23 amino acids at the C-terminus and CΔNSS resulted in cytoplasmic fluorescence, revealing that NSS promotes nuclear transduction despite C-terminal cleavage, and that NSS deficiency leads to nuclear localization. When NSS was fused with green fluorescent protein, it acted more like NES, indicating that additional components are required to mediate the action of NSS (Lin et al., 2007).

### Nuclear Protein Export Machinery

Nuclear export sequences (NES) controlled by chromosome region maintenance 1 (CRM1) govern the active trafficking of several cellular proteins between the nucleus and the cytoplasm (Lee et al., 2020). Amino acids 207–221 of HO-1 share more than 90% isogeny with the documented NES (called NSS) in mice (Lee and Bai 2002; Connor et al., 2003). NES can bind to export receptor CRM1, serving as an exportin in most cases, to form a compound with Ran-GTP, which permits to pass through the nuclear pore (Yan et al., 1998). But this combination can be suppressed by an antimicrobial, Leptomycin-B, which would finally inhibit CRM1-mediated nuclear cytoplasmic transport and consequently increase nuclear HO-1 expression (Yan et al., 1998; Connor et al., 2003). However, there are certain situations that NES motifs may serve as NLS in proteins like UL84 human cytomegalovirus (Lischka et al., 2006). Removal of the assumed NSS could not get rid of the binding of HO-1 to CRM1 entirely, suggesting that other combination area of CRM1 area may exist (Lin et al., 2007). Given the fact that Ran-GTP is not necessary for nuclear translocation of the β-subunit nuclear pore targeting complex, and HO-1 and CRM1 work together, CRM1 is likely to play a role in the nuclear pore bidirectional shuttle (Kose et al., 1997). In addition, oxidative modification can alter NES function and affect NES-CRM1 binding (Velichkova and Hasson 2005). For example, when the NLS-containing protein Nrf2 combines with the NES-dominant protein Keap1, it is retained in the cytoplasm. When NES is oxidatively modified, Nrf2 segregates out of the complex and transferred to nuclei (Velichkova and Hasson 2005).

### The Crosstalk Between Nuclear HO-1 and Signaling Pathways

**FIGURE 2:**
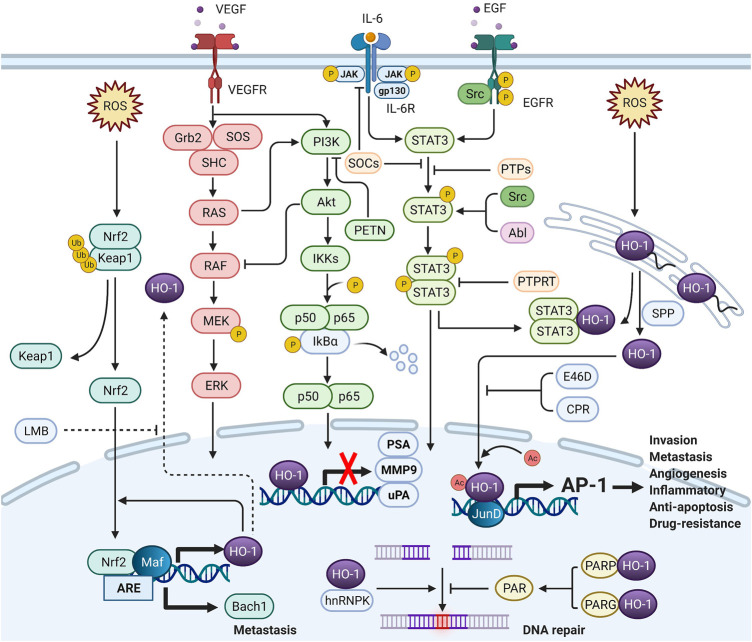
The crosstalk between nuclear HO-1 and signaling pathways (Created with BioRender.com.). In the nucleus, HO-1 interacts with other proteins to alter transcription and translation. HO-1 completely binds to NF-κB and STAT3, thus reducing the activity of the PSA, MMP9 and uPA promoters and mRNA levels. HO-1 also binds to JunD to activate AP-1, increasing tumor aggressiveness. Heterogeneous nuclear ribonucleoprotein K (hnRNPK) has the effect of inhibiting protein translation in the cytoplasm. When hnRNPK and HO-1 migrate to the nucleus together, DNA repair can be initiated. When HO-1 binds to PARP, DNA repair can be blocked. HO-1 reacts with Nrf-2 to enhance Bach-1 production, thus promoting metastasis.

Under cellular stress, nuclear HO-1 could regulate the activities of transcription factors independent of enzymatic activity. There is still no cogent evidence to show that DNA-binding consensus sequence has been found in HO-1 protein (Lin et al., 2007). Nuclear HO-1 might alternatively function as a transcriptional cofactor or as a component of transcriptional protein complex. Although accurate molecular mechanisms remain unclear, the interactome of nuclear HO-1 could provide more insights into the interactions of nuclear HO-1 with signaling pathways (Figure 2).

### Nuclear HO-1 and Nrf2/Bach1 Pathway

Nrf2 is an intracellular transcription factor, also an important factor correlated with the regulation of many antioxidants including HO-1. It frequently gathers in the nuclei of several malignant tumor cells as well as in the nucleus of oxidative stress-mediated injury cells (Moi et al., 1994; Nguyen et al., 2009; Chen et al., 2012; Sporn and Liby 2012; Jaramillo and Zhang 2013; Shelton and Jaiswal 2013). Binding of small Maf proteins and Nrf2 to antioxidant response elements in distal enhancers of HO-1 promoter, leading to its rapid induction in oxidative stress (Ryter and Choi 2002). Heme levels regulate the inhibition of HO-1 induction by Bach1, an antagonist of Nrf2 (Oyake et al., 1996). In the regulatory region of human HO-1 gene, other binding sites have been discovered to regulate the induction of HO-1 under the condition of oxidative stress (Alcaraz et al., 2008). Nrf2, for instance, can induce HO-1 to alleviate the damage caused by oxidant stress. The previous study demonstrated that the specific interaction between HO-1 and Nrf2 could stabilize Nrf2 through GSK3β-mediated phosphorylation and proteolytic degradation, thus enhancing aggregation in nuclei (Rada et al., 2012; Chowdhry et al., 2013; Biswas et al., 2014). Growing NQO1 transcription and glucose-6-phosphate dehydrogenase (G6PDH) activity as well as oxidative damage reducing have been found in cells that overexpression of nuclear HO-1. NQO1 can remove a plethora of oxidants and toxic substances by making use of NAD(P)H (Ross et al., 2000). Given the fact that lower expression of NQO1 and G6PDH were detected in Nrf2-deficient cells, it further demonstrated that protection of the cell against oxidative stress damage was linked to HO-1 nuclear translocation (Biswas et al., 2014). Post-induction activation of Nrf2 mediated by nuclear HO-1 may result in the transcription of certain downstream targets, involving Nrf2 itself, of which transcriptional patterns depend on the antioxidant response elements sequences and the activator protein 1 (AP-1) primary core sequences bound to Nrf2 (Wasserman and Fahl 1997b; a). However, it is far from fully understood how the combination of nuclear HO-1 and Nrf2 mediates the transcriptional priority of NQO1 and G6PDH.

Bach-1-mediated HO-1 downregulation is an underlying mechanism that stabilizes the expression of HO-1 by closing the positive “feed forward” activation of HO-1 genes (Lin et al., 2008). Lignitto et al. have shown the connections between transcriptional signature of Bach1 and poor clinical outcomes in lung cancer patients (Lignitto et al., 2019). Meanwhile, Wiel et al. treated mice inoculated lung cancer with additional N-acetylcysteine or dietary vitamin E, and the upregulation of Bach1 were detected in the cells of the experimental group, which was proved to be related to the promotion of tumor metastasis (Wiel et al., 2019). Previous research verified Bach1 is a crucial physiological transcriptional suppresser of HO-1 through competitively binding to small Maf proteins with Nrf2 to repress Maf-recognition elements (Oyake et al., 1996; Ogawa et al., 2001; Sun et al., 2002). The Nrf2 protein is known to be the main switch for the regulation of the anti-oxidation process in cells, which suggests that the interaction between the Nrf2 and nuclear HO-1 could maintain the stabilization of the latter (Beckner et al., 1990; Biswas et al., 2014). The study reveals that Keap1 loss promotes Nrf2 cumulation as well as Bach1 stabilization through the Nrf2-dependent upregulating of HO-1 (Lignitto et al., 2019). Fbxo22, a substrate receptor of CRL1 complex, was shown to mediate the degradation of Bach1 induced by heme. The activation of Nrf2 could suppress the degradation of Fbxo22-dependent Bach1 *via* inducing the expression of HO-1. Accordingly, pharmacological inhibition of HO-1 could promote Fbxo22-mediated Bach1 degradation, thus inhibiting the process of metastasis. Aside from this, the presence of Bach1 can also help promote the growth of the cancer cells by regulating the transcription of certain genes that are involved in the development of metastasis, such as MMP (Yun et al., 2011; Liang et al., 2012; Lee et al., 2013).

### Nuclear HO-1 and MEK/ERK Pathway

The RAF/MEK/ERK signaling pathway is closely correlated with the proliferation of human normal and cancer cells. Micova et al. suggested that neurotoxic signal is associated with the activation of ERK, but others suggested that ERK activation can trigger neuroprotection during ischemia (Micova et al., 2016). Previous study identified that MEK/ERK signaling pathway has connection with HO-1 expression (Chang et al., 2017). Some studies also indicated that the activation of the ERK pathway could be linked to the expression of HO-1, which could help boost the neuroprotective effects of nerve growth factor (Sun et al., 2017). Furthermore, ERK signaling pathway is also linked to the induction of the nuclear HO-1 by isorhamnetin in C2C12 cells. Isorhamnetin stimulates the Nrf2/HO-1 pathway to activate the intracellular ERK pathway rather than the p38 MAPK or JNK pathways (Choi 2016). MAPK activation has also been linked to HO-1 production during ischemia-reperfusion lung damage (Zhang et al., 2002). However, activation of MAPK signaling pathway is not required for HO-1 autoregulation (Lin et al., 2008).

### Nuclear HO-1 and JAK-STAT3 Pathway

HO-1 protein has previously been shown to transfer to the nuclei and interact with non-canonical transcription factors like STAT3 (Lin et al., 2007). The abnormal activation of androgen receptor (AR) is thought to play a vital role in the tumorigenesis and progression of androgen-dependent prostate cancer (PCa) (Zhu and Kyprianou 2008), and androgens were previously shown to enhance the response of IL-6/STAT3 axis (Ueda et al., 2002; Tam et al., 2007). IL-6 overexpression is constantly detected in PCa patients (Nakashima et al., 2000) and abnormal expression of IL-6 can activate the JAK-STAT signaling pathway and affect tumor growth by autocrine or paracrine loops (Aaronson and Horvath 2002; Culig et al., 2005; Aaronson et al., 2007). The presence of STAT3 protein in the PCa is known to correlate with the development and invasion of cancer. It is also believed that the mechanism by which the HO-1 tumor suppressor is activated may be related to the disruption of STAT3 protein (Ni et al., 2000; Mora et al., 2002). The signaling pathway alters the AR-mediated series of responses (Elguero et al., 2012). PIAS3, a competitive STAT3 inhibitor, is structurally similar to AR and can directly bind to STAT3, reducing AR downstream target gene expression mediated by STAT3 (Yamamoto et al., 2003). The findings *in vivo* and *vitro* showed that enhanced HO-1 expression promotes STAT3 retention in the cytoplasm, and co-immunoprecipitation studies verified that this was mainly due to the direct reciprocal action of these two proteins (Elguero et al., 2012). Meanwhile, authors have emphasized that HO-1 induction in PCa cells could suppress the activation of AR by attenuating the activity of prostate-specific antigen (PSA) promoter as well as the expression level of mRNA. Besides, HO-1 can interfere with STAT3 signaling pathway through regulating the transcriptional activity of STAT3 over the downstream targets, such as uPA, survivin, and cyclin D1 (Elguero et al., 2012). In recent years, there are human diseases that are associated with HO-1/STAT3 signaling axis, including lung injury (Wang et al., 2017), autoimmune disease (Brück et al., 2017), liver ischemia reperfusion injury (Huang et al., 2014), and malaria. Intriguingly, HO-1 appears to regulate STAT3 activity differently depending on the cell type. In keratinocytes, for example, HO-1 was produced by heme to decrease STAT3 activity by activating SHP-156 (Zhang et al., 2016). Curcumin-induced HO-1, on the other hand, increased STAT3 phosphorylation in dendritic cells (Brück et al., 2017).

Furthermore, STAT3 expression profiles like as BCL2 and BCL-XL increase survival (Huynh et al., 2019). In breast cancer cells, HO-1 knockdown increased doxorubicin-induced apoptosis while concurrently downregulating BCL2 and BCL-XL expression (Zhu et al., 2015). HO-1 deletion, on the other hand, hindered HO-1 nuclear migration at the source, interfered with HO-1 protein-STAT3 interaction in the nucleus, and hence suppressed BCL2 and BCL-XL production. More studies are needed since no direct evidence of the link between BCL-2 and nuclear HO-1 translocation has been found.

### Nuclear HO-1 and NF-κB Pathway

NF-κB is a critical nuclear transcription factor that has recently been linked to increased microvascularization and poor outcomes in cancer patients (Ushio-Fukai and Nakamura 2008). In the cytoplasm, the NF-B protein forms a homo/heterodimer of p65 and p50, which binds to inhibitory protein IkB to form an inactive trimeric complex (Gasparian et al., 2002). When the NF-κB dimer separates from the trimer due to the phosphorylation of IkB protein by IkB kinase and the exposure to the localization sequence of the nuclear system, it can enter the nucleus from the cytoplasm and bind to specific sequences on the nuclear DNA, thereby promoting the transcription of related genes, such as CyclinD1, α5β1 integrin (Akalu et al., 2005), MMP9 (Mira et al., 2004; Shukla et al., 2004), VEGF (Huang et al., 2000; Shukla et al., 2004; Kong et al., 2007). When NF-κB was inhibited, downstream pathways linked with blood vessel production and tumor cell proliferation were shown to be down-regulated, which was verified in various PCa models (Huang et al., 2001; Gasparian et al., 2002; Kong et al., 2007). *In vivo* studies also showed that intradermal inoculation with HO-1-stably transfected PC3 cells decreased the invasion of tumors, which was consistent with HO-1 overexpression inhibiting NF-κB activation, inducing IkB accumulation, and reducing IKK mRNA levels (Gueron et al., 2009). In a mouse model of diabetes mellitus and diabetic nephropathy, the HO-1/NF-κB signaling pathway was also established, with reduced expression of Nrf2 and HO-1 and increased expression of NF-κB. And treatment with Apigenin-SLNPs can reverse this, as it inhibits variables linked to proinflammatory cytokine activation, such as IL-6, IL-1, and TNF-α (Gupta et al., 2020; Li et al., 2020).

In addition, some studies demonstrated that HO-1 induction is implicated in the NF-κB pathway and functions as chemotherapy resistance. It has been demonstrated that A1, a member of the BCL family and an NF-κB-dependent anti-apoptotic gene, may be transferred to exhibit anti-apoptotic action (Soares et al., 2001). Chemotherapy resistance in malignancies with HO-1 overexpression might be attributed to enhanced transcription of anti-apoptosis factors *via* the NF-κB pathway. The expression of HO-1 in primary acute myeloid leukemia cells improves after treatment with NF-B inhibitors, suggesting that the NF-κB protein might be involved in HO-1-induced tumor growth (Rushworth et al., 2010). Therefore, combined suppression of NF-κB and HO-1 may become the novel strategy for addressing the thorny problem of treatment resistance in AML. In addition, there are evidences that HO-1 may influence IL-1-induced apoptosis in individuals with intervertebral disc degeneration via the NF-κB signaling pathway (Zhu et al., 2018).

### Associations Between Nuclear HO-1 Expression and Human Diseases

The discovery of HO-1 nuclear localization was regarded as a significant step in the development of human diseases. It has been known that changes in the expression of nuclear HO-1 have been associated with various diseases, involving cancer genesis and progression, neurological diseases, oxidative stress damage and metabolic diseases.

### Cancers

Many studies have shown that the presence of high levels of HO-1 is associated with the development of various cancers (Table 1). The role of this protein in regulating the progression of these diseases could be an important target for the development of drugs. The connections between HO-1 nuclear migration and tumor progression have been previously reviewed (Grochot-Przeczek et al., 2012; Chau 2015; Nitti et al., 2017). In this section, we will provide more evidence supporting the various studies that have shown the link between the nuclear HO-1 and cancers.

**TABLE 1:** Nuclear expression of HO-1 in different cancer types.

Type of cancer	Suggested role	Evidence	References
head and neck squamous cell carcinoma	tumor progression	The rate of nuclear HO-1 in HNSCC was higher than that in nonmalignant tissues and poorly differentiated tumors showed higher percentages of nuclear HO-1	Gandini et al. (2012)
chronic myeloid leukemia	tumor progression and drug resistance	Treated with Ed64 to inhibit HO-1 nuclear translocation can increase imatinib-induced cytotoxicity	Tibullo et al. (2013)
cervical cancer cell	tumor progression	HeLa cells containing excessive t-HO-1H25A was at a rate comparable to cells containing t-HO-1 and was both higher than HO-1H25A and mock cells	Hsu et al. (2015)
H1299 lung cancer line	tumor progression	High expression of t-HO-1 and its mutant form increased the proliferation and migration/invasion of H1299 cells	Hsu et al. (2015)
prostate cancer	carcinogenesis	The relative risk factor for nuclear staining in tumor versus non-tumor parenchyma was 1.8, tumor versus BPH was 3.45, and it was only closely correlated with Gleason score	Maines and Abrahamsson (1996); Sacca et al. (2007)
	tumor suppression	Nuclear HO-1 weakened tumor growth *in vivo* through the NF-κB signaling pathway	Gueron et al. (2009); Ferrando et al. (2011); Elguero et al. (2012)
colorectal cancer	tumor progression	The expression of nuclear HO-1 is transparently higher in less differentiated CRC than well-differentiated CRC.	Yin et al. (2014)

The upregulation of nuclear HO-1 has shown in several types of cancer, including HNSCC (Gandini et al., 2012), chronic myeloid leukemia (Tibullo et al., 2013), cervical cancer cell (Hsu et al., 2015), lung cancer (Hsu et al., 2015), colorectal cancer (Yin et al., 2014), and prostate cancer (Maines and Abrahamsson 1996; Sacca et al., 2007), and are frequently correlates with cancer progression. There is a close connection between increased HO-1 nuclear translocation and tumor differentiation in HNSCC, which has also been shown in the experimental animal model (Gandini et al., 2012). And these results coincide with which that increased expression of nuclear HO-1 are detected in oral epithelial dysplasia as the disease progresses (Lee et al., 2008). Similarly, the fact that nuclear HO-1 expression is clearly higher in less differentiated colorectal cancer (CRC) than in well-differentiated CRC, suggests that nuclear HO-1 overexpression is associated with higher malignant activity (Yin et al., 2014). In cultured Hepa cells, HO-1 nuclear translocation with a truncated form at the C terminus was observed after hypoxic exposure or incubation with heme or H/HPX (Lin et al., 2007). Transfection of HO-1 with small interfering RNA reduces the volume and weight of orthotopic tumor in mice liver (Sass et al., 2008). However, until now, the exact mechanism how nuclear HO-1 actually act on the pathological behavior of human hepatocellular carcinoma has not been fully investigated. As suggested in the studies published by the team of Hsu, a truncated form of HO-1 promotes tumor cell growth, migration, and aggression of cervical carcinoma and lung cancers (Hsu et al., 2015). Besides, the upregulation of nuclear HO-1 in non-small cell lung cancer was related to tumor invasiveness and worse prognosis, further confirmed the anti-apoptotic and cytoprotective effects of nuclear HO-1 in tumor cells (Tsai et al., 2012). Furthermore, they found that nuclear HO-1 is sensitive to acetylation, which is indispensable for nuclear HO-1 to enhance transcriptional activation of AP-1 by interacting with JunD, leading to proliferation, migration, and invasion (Hsu et al., 2017). Interestingly, the dual functions of nuclear HO-1 were found in patients with prostate cancer. Earlier studies have revealed that HO-1 nuclear localization was correlated with prostate carcinogenesis rather than with progression (Sacca et al., 2007). Cigarette smoking induced nuclear translocation of HO-1, however, is likely to be linked with tumor progression rather than initiation (Birrane et al., 2013). In addition, overexpression of HO-1 increased its nuclear localization and inhibited tumor-malignant cell growth in PCa cell lines (Ferrando et al., 2011). It decreased tumor growth *in vivo via* activating the NF-κB signaling pathway and binding to the promoter region of PSA, as well as reducing the expression of angiogenesis and inflammation-related genes (Gueron et al., 2009; Ferrando et al., 2011; Elguero et al., 2012). This difference is probably related to the on/off of specific signaling pathways in different subtypes of prostate cancer.

In addition to directly regulating tumor progression, expression of nuclear HO-1 is also involved in treatment failure of anticancer drugs. For example, chronic myeloid leukemia cells that are resistant to imatinib therapy show more expression of nuclear HO-1, and drug resistance could be reversed by the addition of E64d, a protease inhibitor, or the combination siRNA to imatinib treatable, to impede HO-1 nuclear migration, thus enhancing imatinib-induced cytotoxicity (Tibullo et al., 2013). Furthermore, previously published studies have shown that HO-1 is potentially linked to the chemo-resistance by blocking apoptosis and autophagy of breast cancer cells, and silencing HO-1 can sensitize these resistant cells to doxorubicin (Tan et al., 2015; Zhu et al., 2015).

### Neurological Diseases

Recently, researchers are also exploring the possibility that the presence of HO-1 in the nucleus could contribute to the development of neurological diseases. During postpartum development, the brain is vulnerable to excitotoxic damage (Campisi et al., 1992; Campisi et al., 2003; Ientile et al., 2003). The increased expression of HO-1 protein plays a vital role in neurogenesis and may be associated with the release of oxidative stress-inducing factors by existing amyloid-like microglia (Nakajima et al., 1993). However, the decreased expression of HO-1 may be linked with the transformation of ameboid cells into quiescent microglia in the adult brain (Giulian and Baker 1986). In their study, Li Volti et al. found that in the rat astrocytes, the expression and subcellular distribution of HO-1 were also related to the degree of astrocyte differentiation, suggesting that HO-1 nuclear translocation is underlying mechanism involved in brain development as well as neurodegenerative diseases (Li Volti et al., 2004). Moreover, nuclear HO-1 can significantly improve the spinal cord injury in rats, and the mechanism may be involved in inhibiting endoplasmic reticulum stress and reducing apoptosis of damaged cells (Bi et al., 2020). In general, nuclear translocation of HO-1 is a crucial signaling pathway that preserves cell under the condition of oxidative stress (Gandini et al., 2019). When subjected to oxidative stress, nuclear HO-1 expression increases transcriptional regulation of antioxidant enzymes and resulting in survival advantages (Biswas et al., 2014).

### Oxidative Stress Damages

There is strong evidence supporting the notion that HO-1 could contribute to the regulation of cellular oxidative stress by nuclear migration. In the model of hyperoxic lung injury, nuclear HO-1 can integrate with heterogeneous nuclear ribonucleoprotein K (hnRNPK) and keep it in the nucleus, thereby interacting with β-catenin and regulating the expression of downstream genes (Bomsztyk et al., 2004; Yang et al., 2013). HnRNPK can also bind the 30UTR section of diverse mRNAs to modulate protein transport (Habelhah et al., 2001; Mukhopadhyay et al., 2009). This might explain why postnatal lung is delayed in HO-1 mutant mice (Zhuang et al., 2010). In addition, the capacity of nuclear HO-1 to control the activation of Nrf2 is one of the mechanisms through which it is linked to cytoprotection against oxidative stress. However, there are also conflicting findings, for instance, it has revealed that cytoprotection of HO-1 is intensely correlated with its subcellular distribution as well as expression levels of gene and protein. Their data demonstrated that pneumocytes with high expression level of HO-1 in cytoplasm and nucleus are inclined to continuous abnormal proliferation, on the contrary, the lower expression of HO-1 protect lung tissue from hyperoxia-induced injury by weakening oxidative damage (Namba et al., 2014). This is probably because the overexpression of lung nuclear HO-1 suppressed poly ADP-ribose-dependent adjustment of DNA damage and repair (Koh et al., 2004; Andrabi et al., 2006).

### Metabolic Diseases

HO-1 nuclear translocation has been observed in differentiated brown adipocytes (Giordano et al., 2000). Brown adipose tissue (BAT) is thought to be a unique organ in mammals. BAT can promote the consumption of white fat and regulate glucose homeostasis, as well as insulin sensitivity (Liu et al., 2013; Stanford et al., 2013). Previous studies have found that the BAT content is intensely associated with body mass index, particularly in older persons, indicating a probable character for BAT in body’s metabolism (Cypess et al., 2009). In addition, BAT activity showed an inverse correlation with both body mass index and body fat percentage (van Marken Lichtenbelt et al., 2009). However, it can only be triggered when exposed to cold under normal physiological circumstances, resulting in thermogenic activity and weight reduction (Cannon and Nedergaard 2004). Studies have shown that brown fat could not be activated by cold in some obese patients (van Marken Lichtenbelt et al., 2009). Therefore, activating BAT will become a potentially effective method to treat obesity and diabetes. Giordano et al. found that nuclear HO-1 expression is significantly upregulated after cold exposure, whereas noradrenaline stimulation upregulates its level only in cytosol, but not in the nucleus (Giordano et al., 2000). Therefore, we proposed that HO-1 translocation from cytosol to nuclei may be associated with the activation of brown adipocytes.

## Conclusion and Prospective

Although we have some evidences of the significance of nuclear HO-1 in specific biological and diseased conditions, especially in various types of cancer, many fundamental issues still need to be addressed. This covers a further study of the definite mechanism that how nuclear HO-1 affects cell biology as well as a better understanding of the specific signaling pathway. Indeed, our study indicated that nuclear HO-1 serves on a tumor progressor and inhibiting agent of chemotherapy as well as a tumor suppressor in a variety of cancer types. As described in this review, some studies indicate that the nuclear localization of HO-1 may occur during tumor initiation and tumor expansion. Therefore, a better interpretation of the impact of nuclear HO-1 translocation particularly contributes to comprehension of malignant tumor. In view of the diverse function of HO-1 nuclear translocation, modeling and experimental approaches that incorporate cell heterogeneity and the crosstalk signaling pathway are needed for better estimation of these critical influences. Many of the functions ascribed to HO-1 can be explained by its enzymatic function. However, it is unlikely that HO-1 will have a significant modulator impact on cytoprotection through its by-products if the substrate is not available. Therefore, nonenzymatic functions of HO-1 serving as transcriptional adjustors in nucleus have been of great importance. And this signaling function of the inactive form of HO-1 is probably associated with specific clinical diseases, especially cancers, as reallocation of HO-1 to nucleus has been demonstrated to be associated with cancer progression and metastasis. This may be the mechanism by which HO-1, although it does not have the characteristics of a transcription factor, alters gene expression. These discoveries also imply that HO-1 activity is not indispensable for signaling functions, such as transcription factor activation. In conclusion, further researches of these multiple signaling pathways involved in HO-1 nuclear localization in cell biology probably increase the possibility of healing and translational in a variety of human diseases.

## Abbreviations

HO, Heme oxygenase; CO, Carbon monoxide; sER, Smooth endoplasmic reticulum; HNSCC, Head and neck squamous cell carcinomas; ROS, Reactive oxygen species; NQO1, NADH quinone oxidoreductase 1; TMS, Terminal transmembrane segment; TM, Transmembrane; SPP, Signal peptide peptidase; NLS, Nuclear localization sequence; NSS, Nuclear shuttling sequence; NES, Nuclear export sequences; CRM1, Chromosome region maintenance 1; AP-1, Activator protein 1; AR, Androgen receptor; PCa, Prostate cancer; PSA, Prostate-specific antigen; NF-κB, Nuclear factor-κB; MMP, Proteins of the matrix metalloproteinase; Nrf2, Nuclear factor erythroid 2-related factor 2; GSK3, Glycogen synthase kinase 3; CRC, Colorectal cancer; hnRNPK, Heterogeneous nuclear ribonucleoprotein K; BAT, Brown adipose tissue.
